# Anesthesia With Propofol Sedation Reduces Locoregional Recurrence in Patients With Breast Cancer Receiving Total Mastectomy Compared With Non-Propofol Anesthesia

**DOI:** 10.3389/fonc.2022.708632

**Published:** 2022-03-03

**Authors:** Jiaqiang Zhang, Chia-Lun Chang, Chang-Yun Lu, Ho-Min Chen, Szu-Yuan Wu

**Affiliations:** ^1^ Department of Anesthesiology and Perioperative Medicine, Henan Provincial People’s Hospital, People’s Hospital of Zhengzhou University, Zhengzhou, China; ^2^ Department of Hemato-Oncology, Wan Fang Hospital, Taipei Medical University, Taipei, Taiwan; ^3^ Department of Internal Medicine, School of Medicine, College of Medicine, Taipei Medical University, Taipei, Taiwan; ^4^ Department of General Surgery, Lo-Hsu Medical Foundation, Lotung Poh-Ai Hospital, Yilan, Taiwan; ^5^ Department of Food Nutrition and Health Biotechnology, College of Medical and Health Science, Asia University, Taichung, Taiwan; ^6^ Big Data Center, Lo-Hsu Medical Foundation, Lotung Poh-Ai Hospital, Yilan, Taiwan; ^7^ Division of Radiation Oncology, Lo-Hsu Medical Foundation, Lotung Poh-Ai Hospital, Yilan, Taiwan; ^8^ Department of Healthcare Administration, College of Medical and Health Science, Asia University, Taichung, Taiwan; ^9^ Graduate Institute of Business Administration, College of Management, Fu Jen Catholic University, Taipei, Taiwan; ^10^ Centers for Regional Anesthesia and Pain Medicine, Wan Fang Hospital, Taipei Medical University, Taipei, Taiwan

**Keywords:** propofol, general anesthesia, survival, invasive ductal carcinoma, total mastectomy

## Abstract

**Purpose:**

We examined locoregional recurrence (LRR) in patients with breast invasive ductal carcinoma (IDC) receiving total mastectomy (TM) under propofol-based paravertebral block-regional anesthesia (PB-RA) versus sevoflurane-based inhalational general anesthesia (INHA-GA) without propofol. All-cause death and distant metastasis were secondary endpoints.

**Patients and Methods:**

Patients with breast IDC receiving TM were recruited through propensity score matching and categorized into INHA-GA with sevoflurane and PB-RA with propofol groups. Cox regression analysis was performed to calculate hazard ratios (HRs) and 95% confidence intervals (CIs).

**Results:**

In the multivariate Cox regression analysis, the adjusted HR (aHR; 95% CI) of LRR for the PB-RA with propofol group was 0.52 (0.28–0.96) compared with the INHA-GA with sevoflurane group. The aHRs of LRR for differentiation grade II, grade III, the American Joint Committee on Cancer clinical stage II, stage III, pathological tumor (pT) stage 2, pT stage 3–4, pathological nodal (pN) stage 1, and pN stage 2–3 were 1.16 (1.04–2.08), 1.28 (1.07–2.12), 3.71 (1.82–7.59), 4.67 (1.65–13.18), 1.09 (1.02–1.21), 1.17 (1.03–2.16), 1.10 (1.03–1.33), and 1.22 (1.06–2.41), respectively, compared with differentiation grade I, clinical stage I, pT1, and pN0. The aHR of LRR for adjuvant RT was 0.88 (0.64–0.94) compared with that for no adjuvant RT.

**Conclusion:**

PB-RA with propofol might be beneficial for reducing LRR in women with breast IDC receiving TM compared with INHA-GA without propofol.

## Introduction

Many preclinical studies including *in vivo* or *in vitro* have suggested an association between anesthetic drugs and techniques and the activity and survival of cancer cells; this association can result from changes in the immune response, modulation of the neuroendocrine stress response to surgery, or effects on cancer cell signaling (1–7). However, few studies have reported high-quality clinical outcomes. Most existing clinical studies are retrospective in nature (8–11), and most prospective trials were initially designed to study outcomes other than cancer recurrence (12–14).

Sevoflurane is one of the most widely used volatile anesthetic agents. Sevoflurane exhibited chemoresistance to cisplatin (15) and led to an increased expression of metastasis-related genes (16). By contrast, propofol is the most commonly used intravenous induction agent and is often used for maintaining anesthesia (7). In a laboratory study, propofol exhibited antitumor effects (7). However, investigating the effects of anesthetics, such as sevoflurane and propofol, on patients with cancer in a clinical trial is difficult (17, 18) because patients generally require a combination of anesthetic agents (19, 20). Patients are often managed with either inhalation agents and opioids or propofol as the anesthetic agent and regional anesthesia as the analgesic agent (19, 20). Moreover, performing surgery without providing perioperative pain relief or solely under regional anesthesia to examine the effects of specific anesthetic modalities would be unethical (19, 20). In addition, interpretation of these findings from controversial conclusions in previous studies is limited by heterogeneity resulting from the different extents of surgery, cancer types, and patient characteristics as well as other limitations associated with the retrospective nature of most studies (21). Therefore, conflicting conclusions have been reported in preclinical and clinical studies (1–7, 19, 20).

To address this crucial problem, we chose a consistent extent of surgery (total mastectomy [TM]) for patients with breast invasive ductal carcinoma (IDC), consistent anesthesia (propofol-based paravertebral block-regional anesthesia [PB-RA] vs. sevoflurane-based inhalational general anesthesia [INHA-GA]), and the primary endpoint of locoregional recurrence (LRR) to investigate LRR between INHA-GA without propofol and PB-RA with propofol in patients with breast cancer who underwent TM through propensity score matching (PSM).

## Patients and Methods

### Study Cohorts

This retrospective study was conducted using data from the Health and Welfare Data Center (HWDC) established by Taiwan’s Ministry of Health and Welfare. The HWDC consolidates data gathered by the Taiwanese government from various sources. These data are then deidentified and made available for research purposes based on case-by-case approval. In particular, we used the Taiwan Cancer Registry, which includes the detailed staging and treatment information of patients with cancer, the Cause of Death database, which lists all death certificates issued in Taiwan (22), and the National Health Insurance Research Database, which contains billing information on all National Health Insurance (NHI)-reimbursed examinations, medications, and treatments. We have confident are that no evidence of death is evidence of life, because all death certificates issued is the Government system-specific judgment. A death certificate is required for property inheritance, abandonment of inheritance to the court, burial or cremation. The NHI program has been implemented since 1995 and covers more than 99% of Taiwan’s population.

We established a cohort consisting of female patients with breast IDC by using data from the Taiwan Cancer Registry Database (TCRD), which is maintained by the Collaboration Center of Health Information Application. We enrolled patients who received a diagnosis of IDC between January 1, 2009, and December 31, 2018, and underwent TM. The follow-up duration was from the index date to December 31, 2019. The index date was the date of TM. The mean follow-up duration was 43.3 months (standard deviation [SD], 29.8 months) and 55.9 months (22.6 months) for patients receiving INHA-GA without propofol and those receiving PB-RA with propofol, respectively. The TCRD contains detailed cancer-related information including the clinical or pathological stage (according to the American Joint Committee on Cancer [AJCC], seventh edition), anesthesia modalities, hormone receptor (HR) status, human epidermal growth factor receptor-2 (HER2) status, and radiotherapy (RT) and chemotherapy regimens used (23–27). The study protocols were reviewed and approved by the Institutional Review Board of Tzu-Chi Medical Foundation (IRB109-015-B). Patient diagnoses were confirmed on the basis of pathological data, and patients who received a new diagnosis of breast IDC were confirmed to have no other cancers and no distant metastasis. In the PB-RA with propofol group, propofol was initially used as target-controlled infusion for conscious sedation during paravertebral block and TM (28). The optimal propofol target concentration was ≥0.8 mg/ml at least for the PB-RA with propofol group (29). In the INHA without propofol group, anesthesia was continued with sevoflurane in 100% oxygen at a flow rate of ≥5 L/min in a circle system, and the end-tidal concentration of sevoflurane was maintained at a minimum alveolar concentration of approximately ≥2 (30). Our propofol doses in our study were similar with the previous studies (20, 31). There is no association of the cost of propofol, cost of treatment, and not affected by insurance or decision to in the chose either type of anesthesia. All surgical procedures and propofol cost of treatment for breast cancer were all covered by NHI. Propofol was not used in the INHA-GA group. Other inclusion criteria were age ≥20 years and AJCC clinical stage I–III. Patients with metastasis, missing sex data, age <20 years, nonstandard adjuvant breast RT (contrast with standard adjuvant RT, consisting of irradiation to both the chest wall/whole breast and regional nodes with a minimum of 50 Gy), neoadjuvant chemotherapy, unclear differentiation of tumor grade, missing HR status, missing HER2 status, or unclear pathological tumor, node, and metastasis (TNM) staging were excluded. Adjuvant treatments such as adjuvant RT, adjuvant chemotherapy, hormone therapy, and target therapy were allowed on the basis of National Comprehensive Cancer Network (NCCN) guidelines for breast cancer in Taiwan (32). Furthermore, we excluded patients with unclear surgical procedures, ill-defined nodal surgery, unclear HR status, unclear HER2 status, unknown pathologic TNM stages, unknown American Society of Anesthesiology (ASA) physical status, unclear Charlson comorbidity index (CCI), unclear grade of differentiation, or nonrecorded hospital type (33) (academic center or community hospital) from our cohort. HR positivity was defined as ≥1% of tumor cells demonstrating positive nuclear staining through immunohistochemistry (34) and HER2 positivity was defined as an immunohistochemistry score of 3+ or a fluorescence *in situ* hybridization ratio of ≥2 (33, 35). Finally, we enrolled patients with breast IDC receiving TM under PB-RA with propofol or INHA-GA without propofol during perioperative anesthesia. Comorbidities were assessed using the CCI (36, 37). The CCI has prognostic significance for all-cause death in patients with breast cancer (38, 39). Only comorbidities observed 6 months before the index date were included, and new-onset comorbidities diagnosed within 6 months before the index date were excluded. On the basis of the inclusion criteria, we examined the effects of long-term comorbidities on the survival of patients. Comorbidities were identified according to primary *International Classification of Diseases, Ninth Revision, Clinical Modification* (ICD-9-CM) codes; diseases present at the first admission and those identified more than twice during outpatient visits were included as comorbidities.

### PSM and Covariates

After adjustment for confounders, we used a Cox proportional-hazards model to model time from the index date to LRR (primary endpoint) for patients with IDC receiving TM. To reduce the effects of potential confounders when LRR was compared between different anesthesia groups, PSM was performed. Matching variables used were age, menopausal status, diagnosis year, CCI score, differentiation, AJCC clinical stage, pathological tumor (pT) stage, pathological nodal (pN) stage, ASA physical status, adjuvant chemotherapy, adjuvant RT, HR status, HER2 status, nodal surgery, and hospital level. We matched the cohorts at a ratio of 1:1 by using the greedy method, with age, diagnosis year, menopausal status, CCI score, differentiation, AJCC clinical stage, pT, pN, adjuvant RT, HR status, HER2 status, and nodal surgery completely matched with a propensity score within a caliper of 0.2 (40). Matching is a common technique used for selecting controls with identical background covariates as study participants to minimize differences between individuals that the investigator believes must be controlled. A Cox model was used to regress all-cause death and distant metastasis (DM; secondary endpoints) on different anesthesia statuses, and a robust sandwich estimator was used to account for clustering within matched sets (41). Multivariate Cox regression analysis was performed to calculate hazard ratios (HRs) to determine whether factors such as different anesthesia modalities, age, menopausal status, diagnosis year, CCI score, differentiation, AJCC clinical stage, pT, pN, ASA physical status, adjuvant chemotherapy, adjuvant RT, HR status, HER2 status, nodal surgery, and hospital level are potential independent predictors of all-cause death, LRR, or DM. Potential predictors were controlled for in the analysis (**Table 1**), and LRR was the primary endpoint in both anesthesia groups. All-cause death and DM were the secondary endpoints in our study.

**Table 1:** Demographics of propensity score-matched patients with breast cancer receiving total mastectomy under PB-RA with propofol or INHA-GA without propofol.

		INHA-GA without propofol *N* = 707		PB-RA with propofol *N* = 707		*p*-value
		*n*	(%)	*n*	(%)	
Age (years)	Mean (SD)	56.4	(12.4)	56.1	(12.4)	0.9999
	Median (Q1–Q3)	56	(47-64)	55	(47-64)	
	20–49	236	(33.4)	236	(33.4)	1.0000
	50+	471	(66.6)	471	(66.6)	
Diagnosis year	2009–2013	210	(29.7)	210	(29.7)	1.0000
	2014–2018	497	(70.3)	497	(70.3)	
Menopausal status	Premenopausal	282	(39.9)	282	(39.9)	1.0000
	Postmenopausal	425	(60.1)	425	(60.1)	
CCI scores	0	478	(67.6)	476	(67.3)	0.6530
	1	148	(20.9)	149	(21.1)	
	2+	81	(11.5)	82	(11.6)	
Differentiation	I	68	(9.6)	68	(9.6)	1.0000
	II	486	(68.7)	486	(68.7)	
	III	153	(21.6)	153	(21.6)	
AJCC clinical stage	I	206	(29.1)	206	(29.1)	1.0000
	II	382	(54.0)	382	(54.0)	
	III	119	(16.8)	119	(16.8)	
pT	pT1	269	(38.0)	269	(38.0)	1.0000
	pT2	345	(48.8)	345	(48.8)	
	pT3–4	93	(13.2)	93	(13.2)	
pN	pN0	369	(52.2)	369	(52.2)	1.0000
	pN1	184	(26.0)	184	(26.0)	
	pN2–3	154	(21.8)	154	(21.8)	
ASA physical status	ASA I	400	(56.6)	384	(54.3)	0.5510
	ASA II	167	(23.6)	172	(24.3)	
	ASA III–IV	140	(19.8)	151	(21.4)	
Adjuvant chemotherapy	No	254	(35.9)	243	(34.4)	0.7214
	Yes	453	(64.1)	464	(65.6)	
Adjuvant RT	No	410	(58.0)	419	(59.3)	0.3952
	Yes	297	(42.0)	288	(40.7)	
HR status	No	373	(52.8)	375	(53.0)	0.7520
	Yes	334	(47.2)	332	(47.0)	
HER2 status	No	577	(81.6)	586	(82.9)	0.5149
	Yes	130	(18.4)	121	(17.1)	
Nodal surgery	ALND	510	(72.1)	508	(71.9)	0.8629
	SLNB	197	(27.9)	199	(28.1)	
Hospital level	Academic centers	553	(78.2)	553	(78.2)	1.0000
	Nonacademic	154	(21.8)	154	(21.8)	
Follow-up time, months	Mean (SD)	55.9	(26.6)	43.3	(29.8)	0.7298
All-cause death		79	(11.2)	66	(9.3)	0.0901
Locoregional recurrence		44	(6.2)	27	(3.8)	0.0110
Distant metastasis		82	(11.6)	61	(8.6)	0.0521

IQR, interquartile range; PB-RA, paravertebral block-regional anesthesia; GA, general anesthesia; INHA, inhalational; SD, standard deviation; AJCC, American Joint Committee on Cancer; HER2, Human Epidermal Growth Factor Receptor-2; RT, radiotherapy; ASA, American Society of Anesthesiology; CCI, Charlson comorbidity index; T, tumor; N, nodal; pT, pathological tumor stage; pN, pathological nodal stage; ALND, axillary lymph node dissection; SNLB, sentinel lymph node biopsy.

### Statistics

All analyses were performed using SAS version 9.4 (SAS Institute, Cary, NC, USA). In a two-tailed Wald test, *p* < 0.05 was considered significant. Overall survival (OS), LRR-free survival, and DM-free survival were estimated using the Kaplan–Meier method, and differences between the INHA-GA without propofol and PB-RA with propofol groups were determined using the stratified log-rank test to compare survival curves (stratified according to matched sets) (42).

## Results

### PSM and Study Cohort

The matching process yielded a final cohort of 1,414 patients (707 and 707 in the INHA-GA without propofol and PB-RA with propofol groups, respectively) eligible for further analysis; their characteristics are summarized in **Table 1**. Age distribution was balanced between the two groups (**Table 1**). Menopausal status, diagnosis year, CCI score, differentiation, AJCC clinical stages, pT, pN, hospital level, adjuvant RT, adjuvant chemotherapy, ASA physical status, HR status, HER2 status, and nodal surgery were similar after head-to-head PSM in the two cohorts, and no significant differences were observed in the variables between the two cohorts. The follow-up duration, LRR, DM, or all-cause death was not matched because oncological outcomes were inconsistent between the two groups (**Table 1**). The crude primary endpoint of LRR in women with breast IDC receiving TM under INHA-GA without propofol and PB-RA with propofol varied significantly (*p* = 0.0110; **Table 1**).

### Prognostic Factors for All-Cause Death After Multivariate Cox Regression Analysis

No significant differences in OS were observed in explanatory variables except for age ≥ 50 years, differentiation grade II (moderate differentiation), grade III (poor differentiation), AJCC clinical stage II–III, pT2, pT3–4, pN1, and pN2–3 (**Table 2**). In the multivariate Cox regression analysis, the adjusted HR (aHR; 95% CI) of all-cause death for PB-RA with propofol compared with INHA-GA without propofol was 1.01 (0.68–1.51). The aHRs (95% CIs) of all-cause death for age ≥ 50 years, differentiation grade II, grade III, AJCC clinical stage II, clinical stage III, pT2, pT3–4, pN1, and pN2–3 were 1.64 (1.03–2.62), 2.85 (1.13–7.15), 3.83 (1.48–9.93), 1.42 (1.12–2.45), 1.56 (1.28–3.13), 1.70 (1.07–2.72), 3.06 (1.72–5.43), 1.74 (1.07–2.83), and 3.55 (2.10–6.01), respectively, compared with age < 50 years, differentiation grade 1, AJCC clinical stage I, pT1, and pN0. The aHR of all-cause death for adjuvant chemotherapy was 0.40 (0.27–0.60) compared with no adjuvant chemotherapy.

**Table 2:** Multivariate analysis of all-cause death for propensity score-matched patients with breast cancer receiving total mastectomy under PB-RA with propofol or INHA-GA without propofol.

		All-cause death		
		aHR^*^	(95% CI)	p-value
Anesthesia	INHA-GA	ref		0.9497
	Propofol	1.01	(0.68–1.51)	
Age (years)	20–49	ref		0.0386
	50+	1.64	(1.03–2.62)	
Diagnosis year	2009–2013	ref		0.1900
	2014–2018	0.75	(0.49–1.15)	
Menopausal status	Premenopausal	ref		0.7093
	Postmenopausal	1.09	(0.75–1.54)	
CCI scores	0	ref		0.0807
	1	0.89	(0.54–1.46)	
	2+	1.56	(0.91–2.69)	
Differentiation	I	ref		0.0172
	II	2.85	(1.13–7.15)	
	III	3.83	(1.48–9.93)	
AJCC clinical stage	I	ref		0.0051
	II	1.42	(1.12–2.45)	
	III	1.56	(1.28–3.13)	
pT	pT1	ref		0.0007
	pT2	1.70	(1.07–2.72)	
	pT3–4	3.06	(1.72–5.43)	
pN	pN0	ref		<0.0001
	pN1	1.74	(1.07–2.83)	
	pN2–3	3.55	(2.10–6.01)	
Nodal surgery	ALND	ref		0.3374
	SLNB	1.06	(0.73–1.31)	
ASA	I	ref		0.1308
	II	1.03	(0.62–1.69)	
	III–IV	1.58	(0.93–2.68)	
Adjuvant chemotherapy	Yes	0.40	(0.27–0.60)	<0.0001
Adjuvant RT	Yes	0.96	(0.62–1.49)	0.8469
HR	Positive	0.98	(0.67–1.43)	0.9121
HER2	Positive	1.10	(0.72–1.68)	0.6563
Hospital level	Academic centers	ref		0.2536
	Nonacademic	1.29	(0.83–2.00)	

PB-RA, paravertebral block-regional anesthesia; GA, general anesthesia; INHA, inhalational; aHR, adjusted hazard ratios; CIs, confidence intervals; AJCC, American Joint Committee on Cancer; HR, Hormone Receptor; HER2, Human Epidermal Growth Factor Receptor-2; RT, radiotherapy; ASA, American Society of Anesthesiology; CCI, Charlson comorbidity index; T, tumor; N, nodal; pT, pathological tumor stage; pN, pathological nodal stage; ALND, axillary lymph node dissection; SNLB, sentinel lymph node biopsy; ref, reference group.

*All covariates mentioned in **Table 2** were adjusted.

### Prognostic Factors for LRR After Multivariate Cox Regression Analysis

The aHR (95% CI) of LRR for the PB-RA with propofol group was 0.52 (0.28–0.96) compared with the INHA-GA without propofol group (**Table 3**). The aHRs of LRR for differentiation grade II, grade III, clinical stage II, stage III, pT2, pT3–4, and pN2–3 were 1.16 (1.04–2.08), 1.28 (1.07–2.12), 3.71 (1.82–7.59), 4.67 (1.65–13.18), 1.09 (1.02–1.21), 1.17 (1.03–2.16), 1.10 (1.03–1.33), and 1.22 (1.06–2.41), respectively, compared with differentiation grade I, clinical stage I, pT1, and pN0. The aHR of LRR for adjuvant RT was 0.88 (0.64–0.94) compared with that for no adjuvant RT.

**Table 3:** Multivariate analysis of locoregional recurrence for propensity score-matched patients with breast cancer receiving total mastectomy under PB-RA with propofol or INHA-GA without propofol.

		LRR		
		aHR^*^	(95% CI)	p-value
Anesthesia	INHA-GA	ref		0.0365
	Propofol	0.52	(0.28–0.96)	
Age (years)	20–49	ref		0.9111
	50+	0.97	(0.55–1.72)	
Diagnosis year	2009–2013	ref		0.2513
	2014–2018	1.13	(0.90–3.75)	
Menopausal status	Premenopausal	Ref		0.7081
	Postmenopausal	0.81	(0.71–1.30)	
CCI scores	0	ref		0.1309
	1	1.04	(0.80–1.06)	
	2+	1.07	(0.76–2.49)	
Differentiation	I	ref		0.0099
	II	1.16	(1.04–2.08)	
	III	1.28	(1.07–2.12)	
AJCC clinical stage	I	ref		0.0012
	II	3.71	(1.82–7.59)	
	III	4.67	(1.65–13.18)	
pT	pT1	ref		0.0260
	pT2	1.09	(1.02–1.21)	
	pT3–4	1.17	(1.03–2.16)	
pN	pN0	ref		0.0022
	pN1	1.10	(1.03–1.33)	
	pN2–3	1.22	(1.06–2.41)	
Nodal surgery	ALND	ref		0.3066
	SLNB	1.55	(0.72–3.36)	
ASA	I	ref		0.2221
	II	1.16	(0.57–2.38)	
	III-IV	1.89	(0.90–3.96)	
Adjuvant chemotherapy	Yes	1.26	(0.71–2.25)	0.4343
Adjuvant RT	Yes	0.88	(0.64–0.94)	0.0413
HR	Positive	0.88	(0.68–3.28)	0.2252
HER2	Positive	1.64	(0.89–3.02)	0.1103
Hospital level	Academic centers	ref		0.1078
	Nonacademic	0.56	(0.28–1.13)	

PB-RA, paravertebral block-regional anesthesia; GA, general anesthesia; INHA, inhalational; aHR, adjusted hazard ratios; CIs, confidence intervals; AJCC, American Joint Committee on Cancer; HR, Hormone Receptor; HER2, Human Epidermal Growth Factor Receptor-2; RT, radiotherapy; ASA, American Society of Anesthesiology; CCI, Charlson comorbidity index; T, tumor; N, nodal; pT, pathological tumor stage; pN, pathological nodal stage; ALND, axillary lymph node dissection; SNLB, sentinel lymph node biopsy; ref, reference group.

*All covariates mentioned in **Table 2** were adjusted.

### Prognostic Factors for DM After Multivariate Cox Regression Analysis

The aHR (95% CI) of DM for the PB-RA with propofol group was 0.74 (0.49–1.10) compared with the INHA-GA without propofol group (**Table 4**). The aHRs of DM for clinical stage II, stage III, pT2, pT3–4, pN1, pN2–3, and HER2 positivity were 1.15 (1.06–2.46), 1.35 (1.12–2.92), 1.12 (1.02–2.21), 2.01 (1.12–3.59), 1.24 (1.11–2.29), 2.11 (1.22–3.64), and 2.06 (1.07–3.52), respectively, compared with clinical stage I, pT1, pN0, and HER2 negativity. The aHR of DM for adjuvant chemotherapy was 0.70 (0.46–0.96) compared with that for no adjuvant chemotherapy.

**Table 4:** Multivariate analysis of distant metastasis for propensity score-matched patients with breast cancer receiving total mastectomy under PB-RA with propofol or INHA-GA without propofol.

		DM		
		aHR*	(95% CI)	p-value
Anesthesia	INHA-GA	ref		0.1369
	Propofol	0.74	(0.49–1.10)	
Age (years)	20–49	ref		0.7548
	50+	0.94	(0.62–1.41)	
Diagnosis year	2009–2013	ref		0.2296
	2014–2018	0.77	(0.50–1.18)	
Menopausal status	Premenopausal	ref		0.4711
	Postmenopausal	0.79	(0.68–1.51)	
CCI scores	0	ref		0.8673
	1	0.88	(0.54–1.43)	
	2+	0.90	(0.47–1.72)	
Differentiation	I	ref		0.7573
	II	1.26	(0.62–2.54)	
	III	1.33	(0.63–2.82)	
AJCC clinical stage	I	ref		0.0089
	II	1.15	(1.06–2.46)	
	III	1.35	(1.12–2.92)	
pT	pT1	ref		0.0015
	pT2	1.12	(1.02–2.21)	
	pT3–4	2.01	(1.12–3.59)	
pN	pN0	ref		0.0073
	pN1	1.24	(1.11–2.29)	
	pN2–3	2.11	(1.22–3.64)	
Nodal surgery	ALND	ref		0.2283
	SLNB	1.09	(0.88–3.25)	
ASA	I	ref		0.9537
	II	1.07	(0.59–1.58)	
	III–IV	1.12	(0.62–1.80)	
Adjuvant chemotherapy	Yes	0.70	(0.46–0.96)	0.0157
Adjuvant RT	Yes	0.81	(0.52–1.26)	0.3475
HR	Positive	0.96	(0.73–1.55)	0.7624
HER2	Positive	2.06	(1.07–3.52)	<0.0001
Hospital level	Academic centers	ref		0.4898
	Nonacademic	0.85	(0.53–1.36)	

PB-RA, paravertebral block-regional anesthesia; GA, general anesthesia; INHA, inhalational; aHR, adjusted hazard ratios; CIs, confidence intervals; AJCC, American Joint Committee on Cancer; HR, Hormone Receptor; HER2, Human Epidermal Growth Factor Receptor-2; RT, radiotherapy; ASA, American Society of Anesthesiology; CCI, Charlson comorbidity index; T, tumor; N, nodal; pT, pathological tumor stage; pN, pathological nodal stage; ALND, axillary lymph node dissection; SNLB, sentinel lymph node biopsy; ref, reference group.

*All covariates mentioned in **Table 2** were adjusted.

## Discussion

Most existing clinical studies were retrospective in nature or included a small sample, and meta-analyses included heterogeneous cancers, surgical techniques, patient populations, and follow-up (8, 9, 11, 16). Multiple factors can be responsible for differences in study findings; for instance, the characteristics and treatments varied among patients with breast IDC in clinical studies, whereas fixed conditions were examined in preclinical studies (22–26, 43–45). Factors affecting breast cancer prognosis are diverse and complex (43–45). For example, adjuvant chemotherapy is indicated for women with advanced pathological stages of breast IDC receiving breast surgery (46, 47); however, no adjuvant chemotherapy was administered in preclinical studies (1–7). Clinical covariates including molecular status (HR or HER2 status) and adjuvant treatment (adjuvant RT or chemotherapy) might result in inconsistent findings in preclinical and clinical studies (1–7, 22–26, 43–45). The only published randomized controlled trial (RCT) including breast-conserving surgery (BCS) or TM for breast cancer showed that the administration of INHA-GA without propofol or PB-RA with propofol exerted no effect on the primary endpoint of cancer recurrence including LRR and DM in patients with breast cancer (20). Moreover, the findings of this RCT are different from those of preclinical studies (1–7). Thus, to address these problems, we included LRR as the primary endpoint and performed PSM to control for all potential covariates in this study with the consistent surgical procedure.

The novelty of our study is the inclusion of LRR as the primary endpoint. No study has included LRR as a study endpoint. We controlled for all the potential covariates of LRR (**Table 1**) and observed no bias between the INHA-GA without propofol and PB-RA with propofol groups through PSM. Additionally, the various extent of surgery might be associated with different hypoxia time related with local recurrence (48, 49). Thus, in our study we maintain a consistent surgical procedure (all patients receiving TM) for breast IDC patients. Our results revealed that patients with breast IDC receiving TM under PB-RA with propofol had a significantly decreased risk of LRR compared with those receiving TM under INHA-GA (sevoflurane) without propofol (**Table 3**). A similar benefit was not observed for OS, possibly because adjuvant treatments might have masked the benefits of PB-RA with propofol; studies with longer follow-up duration should be conducted to examine the effect on OS. In addition, the proportion of patients who developed LRR in our study was small (3.8% and 6.2% for non-propofol and propofol groups, respectively); a larger sample size would be necessary to examine OS. However, our study is the first to investigate the effect of the administration of INHA-GA without propofol or PB-RA with propofol on LRR in patients with breast IDC receiving TM. Our findings for LRR are different from those reported by Sessler et al. who included DM and LRR together to examine cancer recurrence (20). Moreover, to maintain a consistent extent of surgery, we enrolled patients who received TM only and matched them at a ratio of 1:1 by using the greedy method (**Table 1**). In theory, the consistent time and the same extent of surgery related with similar levels of hypoxia (49) could be more consistent between the two anesthesia techniques in our study than Sessler et al.’s study (20). Tissue hypoxia causes an upregulated expression of the transcription factor hypoxia-inducible factor 1-alpha, which is crucial for the promotion of cellular pathways for angiogenesis, cell proliferation, and metastasis (48). Moreover, preclinical studies have reported that propofol exhibits the anticancer property by exerting an immune effect (4, 50, 51). Patients receiving PB-RA with propofol demonstrated an increased level of immune cell infiltration into the breast cancer tissue, an increased level of cancer cell apoptosis, and preserved cytotoxicity of natural killer cells (4, 50, 51). The advantages of PB-RA with propofol observed in preclinical studies were reproduced in our clinical study through head-to-head PSM. Our clinical study indicated differentiation grade II–III, clinical stage II–III, pT2, pT3–4, and pN2–3 as independent poor prognostic factors of LRR; this finding is compatible with those of previous clinical studies (22–26) Adjuvant RT reduced the risk of LRR in patients with breast IDC receiving TM (**Table 3**); this result is also in agreement with that of a previous clinical study (52).

In our study, we examined OS (the secondary endpoint) in patients with breast IDC receiving TM under INHA-GA without propofol and PB-RA with propofol (**Table 2**). We observed that the administration of INHA-GA without propofol or PB-RA with propofol did not exert any effect on the OS of these patients; this finding is compatible with those of previous clinical studies (**Table 2**) (8, 11, 53). All existing studies examining the endpoint of OS were retrospective in nature and included a small sample size, heterogeneous cancers, various surgical techniques, different patient populations, and short follow-up durations (8, 9, 11, 16). A meta-analysis conducted in 2014 found no difference in OS among patients with breast, prostate, colon, and gastroesophageal cancers who received general epidural anesthesia versus GA alone (8). Similarly, in 2017, a meta-analysis of 28 studies (retrospective, observational, and randomized) reported that OS was similar in patients with various cancers who underwent surgery under RA with or without GA and those who underwent surgery under GA alone (11). A meta-analysis of 10 retrospective studies including approximately 13,760 patients who underwent radical prostatectomy for cancer found that RA with or without GA was associated with improved OS but similar cancer recurrence compared with GA alone (9). Furthermore, a meta-analysis suggested that RA was associated with improved OS, particularly in patients with colorectal cancer, as well as a reduced risk of cancer recurrence (10). Therefore, conflicting results have been reported in clinical studies including different cancer types, extents of surgery, and adjuvant treatments (8–11, 16). The inconsistency in the results of clinical and preclinical studies might be attributed to the use of different therapeutic modalities, such as adjuvant chemotherapy, hormone therapy, and tyrosine kinase inhibitors, and different surgical procedures, which might have masked the effects of different anesthesia techniques (RA with propofol or sevoflurane-based INHA-GA) on patients’ OS (22–26, 43–45). By contrast, the findings of multivariate analysis performed in our study indicated that old age, moderate-poor differentiation (grade II–III) (54), clinical stage II–III, pT2, pT3–4, pN1, and pN2–3 were independent poor prognostic factors for all-cause death; this finding is compatible with those of previous clinical studies (20, 22–26). Adjuvant chemotherapy was associated with better OS in patients with breast IDC receiving TM (**Table 2**); this finding is also in accordance with those of previous clinical studies (46, 47, 52). Because the trend of oncological outcomes and prognostic factors for OS in our study was similar to that reported in other studies (20, 22–26, 46, 47, 52, 54–56), the effect of the administration of INHA-GA without propofol or PB-RA with propofol on oncological outcomes (OS, LRR, and DM) in patients with IDC receiving TM might truly exist in real-world clinical practice, although clinical outcomes might vary for different molecular breast types, adjuvant treatments, or extents of surgery. In the current study, most confounding factors like molecular breast types, adjuvant treatments, or extents of surgery (BCS or TM) were consistent or adjusted in our analysis.

As shown in **Table 4**, we observed that the risk of DM was not associated with the administration of INHA-GA without propofol or PB-RA with propofol in patients with IDC receiving TM; this finding differs from those of previous preclinical studies (1–7). Although many preclinical studies have reported that volatile anesthetics can enhance metastasis, such as by exerting direct survival-enhancing effects on cancer cells, suppressing immune cell functions, and killing tumor cells (2–4, 51), no association of DM with the administration of INHA-GA without propofol or PB-RA with propofol in patients with breast IDC receiving TM was observed in our clinical study. In laboratory studies, propofol exhibited antitumor effects by directly regulating key ribonucleic acid pathways and signaling in cancer cells (7). In addition, propofol exerts anti-inflammatory and antioxidative effects (1, 6, 50), which may protect against perioperative immune suppression. Although many preclinical studies have shown that propofol might inhibit cancer metastasis and INHA-GA can enhance cancer metastasis (1–4, 6, 50, 51), these phenomena were not observed in our study (**Table 4**). This difference might be attributed to the use of different adjuvant treatments and the inclusion of various breast cancer molecular types that might have obscured the effects of propofol and sevoflurane (43–45). However, other independent poor or better prognostic factors such as clinical stage II–III, pT2, pT3–4, pN2–3, HER2 positivity, and adjuvant chemotherapy determined in this study are compatible with those observed in previous clinical studies (22–26). **Supplementary Figures 1A–C** present survival curves for OS and LRR-free and DM-free survival obtained using the Kaplan–Meier method for the propensity score-matched cohort of patients with breast IDC receiving TM under PB-RA with propofol or INHA-GA without propofol. The crude LRR-free survival without adjustment for PB-RA with propofol was not significantly longer than that for INHA-GA without propofol for all patients with breast IDC receiving TM (*p* = 0.1430).

The strength of our study is that this is the first and largest cohort study to estimate the primary endpoint of LRR for patients with breast IDC receiving TM under INHA-GA without propofol and PB-RA with propofol. The covariates between the two anesthesia techniques were homogenous for women with breast IDC receiving TM; no selection bias was observed for the two anesthesia techniques through PSM (**Table 1**). No study has examined the effect of PB-RA with propofol on LRR in patients with breast cancer receiving TM, and all prognostic factors including clinical and pathologic stages and molecular types were evaluated. Poor prognostic factors for OS, LRR, or DM determined in patients with breast cancer receiving TM in the present study, namely, moderate-poor differentiation, advanced clinical stages, advanced pathologic TN stages, HER2 positivity, adjuvant RT, and adjuvant chemotherapy (**Tables 2**–**4**), are similar to those reported in previous studies (57–61). In patients with breast IDC receiving TM, adjuvant RT reduced the risk of LRR and adjuvant chemotherapy reduced the risk of DM. However, PB-RA with propofol in patients with breast IDC receiving TM was beneficial only for LRR instead of all-cause death and DM. This is the first study to show that PB-RA with propofol reduced the risk of LRR. Previous studies did not focus on recurrence; thus, LRR and DM could not be distinguished (20, 57–65). Our study is the first to examine the effects of INHA-GA without propofol or PB-RA with propofol on LRR and DM individually instead of breast cancer recurrence including LRR and DM. Our findings should be considered in future clinical practice and prospective clinical trials.

This study has some limitations. First, because all patients with breast IDC were enrolled from an Asian population, the corresponding ethnic susceptibility compared with the non-Asian population remains unclear; hence, our results should be cautiously extrapolated to non-Asian populations. However, no evidence has demonstrated differences in oncological outcomes between Asian and non-Asian patients with breast IDC receiving TM. Second, recently, the propensity score could be currently recommended as a standard tool for investigators trying to estimate the effects of intervention in studies where any potential bias may exist. Although the main advantage of the propensity score methodology is in its contribution to the more precise estimation of intervention response, PSM cannot control for factors not accounted for in the model and is predicated on an [explicit selection bias] of those whom could be a match (i.e., those who could not be matched are not part of the scope of inference). Third, the diagnoses of all comorbid conditions were based on ICD-9-CM codes. Nevertheless, the Taiwan Cancer Registry Administration randomly reviews charts and interviews patients to verify the accuracy of diagnoses, and hospitals with outlier chargers or practices may be audited and subsequently be heavily penalized if malpractice or discrepancies are identified. Accordingly, to obtain crucial information regarding population specificity and disease occurrence, a large-scale randomized trial comparing carefully selected patients undergoing suitable extent of surgery, consistent molecular types, and treatments is essential. Finally, the Taiwan Cancer Registry database does not contain information regarding dietary habits, lifestyle factors, socioeconomic status, or body mass index, all of which may be risk factors for LRR or mortality. However, considering the magnitude and statistical significance of the observed effects in this study, these limitations are unlikely to affect the conclusions.

## Conclusions

PB-RA with propofol might be beneficial in reducing LRR in women with breast IDC receiving TM compared with INHA-GA without non-propofol. INHA-GA without propofol or PB-RA with propofol was not associated with the risk of OS or DM in patients with breast IDC receiving TM.

## Data Availability Statement

The datasets presented in this article are not readily available because we used data from the National Health Insurance Research Database (NHIRD) and Taiwan Cancer Registry database. The authors confirm that, for approved reasons, some access restrictions apply to the data underlying the findings. The data utilized in this study cannot be made available in the manuscript, the **Supplementary Material**, or in a public repository due to the “Personal Information Protection Act” executed by Taiwan’s government, starting from 2012. Requests for data can be sent as a formal proposal to obtain approval from the ethics review committee of the appropriate governmental department in Taiwan. Specifically, links regarding contact info for which data requests may be sent to are as follows: http://nhird.nhri.org.tw/en/Data_Subsets.html#S3 and http://nhis.nhri.org.tw/point.html. Requests to access the datasets should be directed to http://nhird.nhri.org.tw/en/Data_Subsets.html#S3 and http://nhis.nhri.org.tw/point.html.

## Ethics Statement

Our study protocols were reviewed and approved by the Institutional Review Board of Tzu-Chi Medical Foundation (IRB109-015-B). Written informed consent for participation was not required for this study in accordance with the national legislation and the institutional requirements.

## Author Contributions

Conception and Design: JZ, C-LC, C-YL, H-MC, and S-YW. Collection and Assembly of Data: H-MC and S-YW. Data Analysis and Interpretation: JZ, C-LC, and S-YW. Administrative Support: S-YW. Manuscript Writing: JZ, C-LC, and S-YW. Final Approval of Manuscript: All authors. All authors contributed to the article and approved the submitted version.

## Funding

Lo-Hsu Medical Foundation, LotungPoh-Ai Hospital, supports Szu-Yuan Wu’s work (Funding Number: 10908, 10909, 11001, 11002, 11003, 11006, and 11013).

## Conflict of Interest

The authors declare that the research was conducted in the absence of any commercial or financial relationships that could be construed as a potential conflict of interest.

## Publisher’s Note

All claims expressed in this article are solely those of the authors and do not necessarily represent those of their affiliated organizations, or those of the publisher, the editors and the reviewers. Any product that may be evaluated in this article, or claim that may be made by its manufacturer, is not guaranteed or endorsed by the publisher.

## Supplementary Material

Supplementary Figure 1
**(A)** Kaplan–Meier overall survival curves of propensity score–matched patients with breast cancer receiving total mastectomy under PB-RA with propofol or INHA-GA without propofol. **(B)** Kaplan–Meier locoregional recurrence-free survival curves of propensity score–matched patients with breast cancer receiving total mastectomy under PB-RA with propofol or INHA-GA without propofol. **(C)** Kaplan–Meier distant metastasis-free survival curves of propensity score–matched patients with breast cancer receiving total mastectomy under PB-RA with propofol or INHA-GA without propofol.

## Abbreviations

OS, overall survival; LRR, locoregional recurrence; DM, distant metastasis; IDC, invasive ductal carcinoma; TM, total mastectomy; PB-RA, paravertebral block-regional anesthesia; GA, general anesthesia; INHA, inhalational; HR, hazard ratio; aHR, adjusted hazard ratio; CI, confidence interval; RCT, randomized controlled trial; PSM, propensity score matching; TCRD, Taiwan Cancer Registry database; SD, standard deviation; AJCC, American Joint Committee on Cancer; HR, Hormone Receptor; HER2, Human Epidermal Growth Factor Receptor-2; RT, radiotherapy; ASA, American Society of Anesthesiology; CCI, Charlson comorbidity index; ICD-9-CM, International Classification of Diseases, Ninth Revision, Clinical Modification; TNM, Tumor, Node, and Metastasis; T, tumor; N, nodal; pT, pathological tumor stage; pN, pathological nodal stage; NCCN, National Comprehensive Cancer Network; ALND, axillary lymph node dissection; SLNB, sentinel lymph node biopsy.
