# Supplementary figures and images for: Anesthesia With Propofol Sedation Reduces Locoregional Recurrence in Patients With Breast Cancer Receiving Total Mastectomy Compared With Non-Propofol Anesthesia
Source: Front Oncol. 2022 Mar 3;12:708632. doi: 10.3389/fonc.2022.708632 (PMC8927654; doi:10.3389/fonc.2022.708632)

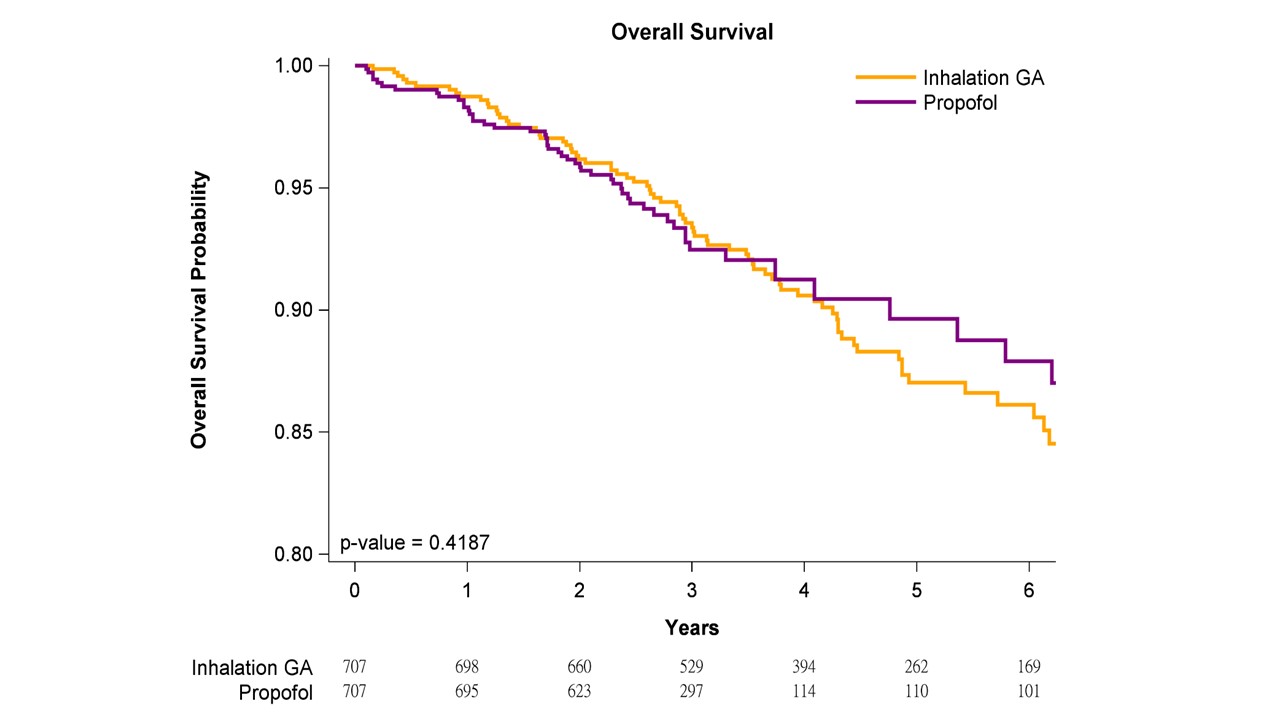

Supplement: Supplementary Figure 1 — (A) Kaplan–Meier overall survival curves of propensity score–matched patients with breast cancer receiving total mastectomy under PB-RA with propofol or INHA-GA without propofol. (B) Kaplan–Meier locoregional recurrence-free survival curves of propensity score–matched patients with breast cancer receiving total mastectomy under PB-RA with propofol or INHA-GA without propofol. (C) Kaplan–Meier distant metastasis-free survival curves of propensity score–matched patients with breast cancer receiving total mastectomy under PB-RA with propofol or INHA-GA without propofol. [file Image_1.jpg]

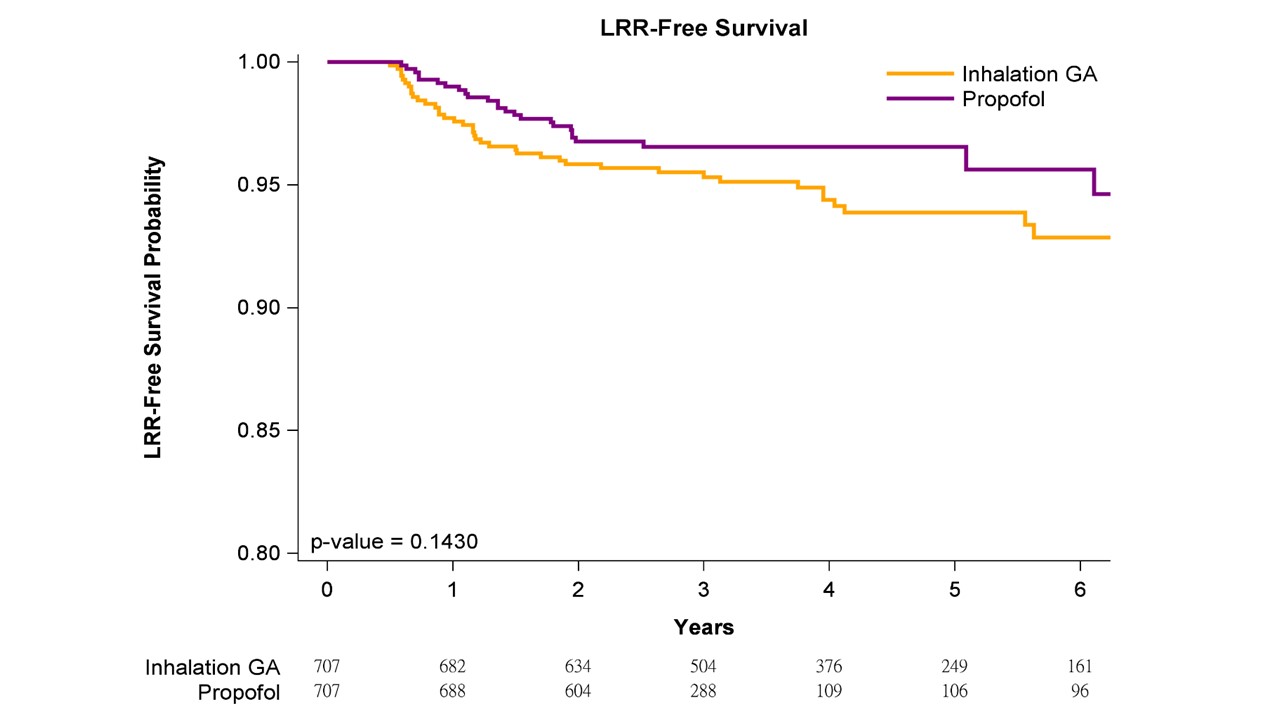

Supplement: Supplementary file 2 [file Image_2.jpg]

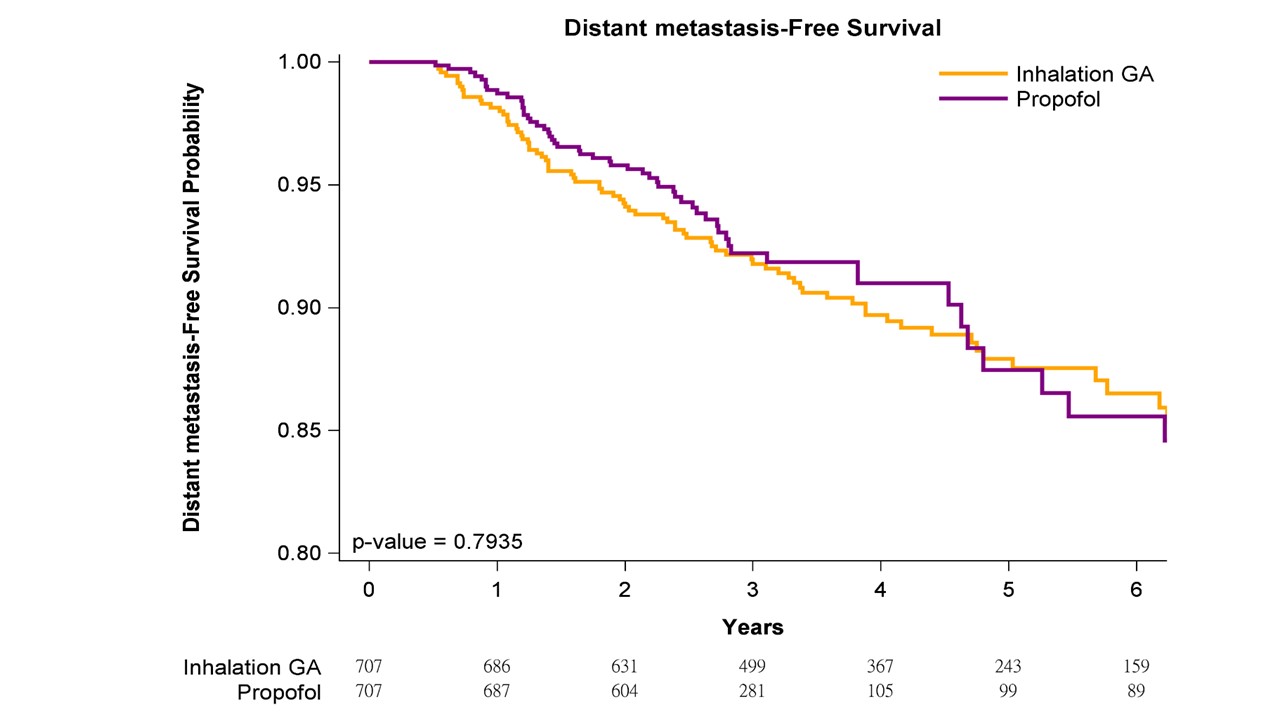

Supplement: Supplementary file 3 [file Image_3.jpg]
